# Parental Intake of Eicosapentaenoic and Docosahexaenoic Acids in a Diverse, Urban City in the United States Is Associated with Indicators of Children’s Health Potential

**DOI:** 10.3390/nu17203277

**Published:** 2025-10-18

**Authors:** Daniel T. Robinson, Marie E. Heffernan, Anne Bendelow, Carly G. Menker, Mia Casale, Tracie Smith, Matthew M. Davis, Susan E. Carlson

**Affiliations:** 1 Department of Pediatrics, Northwestern University Feinberg School of Medicine, Chicago, IL 60611, USA; 2 Ann & Robert H. Lurie Children’s Hospital of Chicago, Chicago, IL 60611, USA; 3 Mary Ann & J. Milburn Smith Child Health Outcomes, Research, and Evaluation Center, Stanley Manne Children’s Research Institute, Ann & Robert H. Lurie Children’s Hospital of Chicago, Chicago, IL 60611, USA; 4 Data Analytics and Reporting, Ann & Robert H. Lurie Children’s Hospital of Chicago, Chicago, IL 60611, USA; 5 Nemours Children’s Health, Wilmington, DE 19803, USA; 6 Department of Dietetics and Nutrition, University of Kansas Medical Center, Kansas City, KS 66103, USA

**Keywords:** parental dietary intake, omega-3 fatty acids, eicosapentaenoic acid, docosahexaenoic acid, seafoods, preterm birth, food frequency questionnaire

## Abstract

**Background/Objectives:** Parents achieving recommended eicosapentaenoic (EPA) and docosahexaenoic (DHA) acid intake can improve the health of parents and their children. Evidence links higher DHA intake to lower preterm birth (PTB) risk. With parental intake poorly defined, the objective is to characterize EPA and DHA intake by parents with children in households in a diverse, urban city. **Methods:** Parents with ≥1 child in the household completed a validated seven-question food frequency questionnaire to assess consumption of foods contributing most to EPA and DHA intake in American diets during the cross-sectional Voices of Child Health in Chicago Panel Survey (May–July 2022). Female respondents reported prior PTB. Home/residence information was linked to the Childhood Opportunity Index (COI). Multivariable linear regression and survey-weighted models evaluated parental characteristics associated with EPA+DHA intake. Pairwise comparisons estimated intake differences (mean (SE)) among groups. **Results:** Chicago parents (n = 1057) reported lower-than-recommended EPA+DHA intake and mothers consumed less compared to fathers (difference: 27.1 (11.4) mg/d; *p* = 0.02). Prior PTB was associated with lower EPA+DHA intake, yet DHA-containing supplement use, which occurred in ~25% of parents, was associated with higher intake (*p* < 0.05). Lower household income and a lower COI were associated with lower intake while parental race and ethnicity categories were also associated with intake (all *p* < 0.05); intake differed for mothers and fathers based on Black race and Hispanic ethnicity categories. **Conclusions:** The findings suggest that efforts aimed at improving parental EPA+DHA intake to improve the health of families should account for multidimensional influences on household food choices.

## 1. Introduction

The most recent Dietary Guidelines for Americans recommend seafood intake, an important source of omega-3 long-chain polyunsaturated fatty acids (PUFAs), across the lifespan [1]. There is strong evidence that intake of specific omega-3 PUFA, eicosapentaenoic acid (EPA; 20:5n-3), and docosahexaenoic acid (DHA; 22:6n-3) reduces preterm birth (PTB) and early PTB (<34 weeks of gestation) [2,3,4]. Moreover, recent reviews link seafood intake during pregnancy and childhood to positive childhood neurodevelopmental outcomes [5,6]. Recommendations of EPA and DHA intake for adults are grounded in evidence of cardiovascular and cognitive benefits [7,8]. Common mechanistic pathways for benefit may be through reduction in inflammation and providing higher concentrations of these PUFAs to the central nervous system [9,10].

Seafoods, particularly oily fish such as salmon and tuna, have the highest concentrations of EPA and DHA. Chicken and eggs are other foods with DHA but contain much smaller amounts. Unless seafood is consumed, diets are typically low in EPA and DHA. It is known that EPA and DHA intake by American children is low [11,12]. Reported intake in adults is also lower than the recommended average daily intake of at least 250 mg of combined EPA plus DHA [1,7,11,13,14,15]. However, EPA and DHA intake specific to parents with children living at home is not defined. Parental dietary habits influence the developing eating habits of their children, with intake of foods categorized as “healthy” more strongly correlated between parents and children than foods categorized as “unhealthy” [16]. The relationship between healthy eating by parents and their children typically compares intake of whole foods or dietary patterns rather than individual nutrients. To improve EPA and DHA intake by children, it is important to understand what contributes to parental intake of foods that contain EPA and DHA.

The objective of this study was to assess EPA and DHA intake by parents living in a diverse, urban city and identify sociodemographic conditions related to the EPA and DHA intake of parents. Furthermore, given the direct relevance of PTB to child health and our standing interest in the role of DHA in this outcome, we also aimed to define intake in mothers who previously had a PTB. We hypothesized that parental average EPA and DHA intake would be lower than 250 mg per day and that sociodemographic characteristics of households and neighborhood communities [17] would be related to intake.

## 2. Methods

Parents from all 77 neighborhoods in Chicago (n = 1057) provided responses to a food frequency questionnaire (FFQ) administered via the web during May–July 2022 for a cross-sectional assessment of EPA and DHA intake. The FFQ asked about consumption of foods that contribute most to EPA and DHA intake in diets of Americans as well as supplement use [18]. Depending on the foods assessed, respondents reported consumption over the past two months or weekly consumption (Supplemental Material). Three of the seven questions asked about intake of seafoods which were grouped by similarity in DHA content. Respondents first answered whether they had consumed any seafoods within that group and then reported consumption frequency for any seafoods in that group. Consumption responses were then converted into an estimated daily intake (mg/d). Parents also reported whether they took DHA-containing supplements. Correlations between the FFQ’s response-based estimated intake of the common food sources of EPA and DHA and red blood cell DHA concentrations as a biomarker of nutrient status previously occurred in 1368 US pregnant women [19,20]. This FFQ was also shown to be valid whether responses occurred through self-report or staff-administered surveys [18].

### 2.1. Survey Administration

The FFQ was a specific module administered within the Voices of Child Health in Chicago (VOCHIC) Parent Panel Survey, a survey of parents from Chicago on topics related to child and family health. For inclusion, parents were ≥18 years old and the parent or guardian of at least one child between 0 and 17 years old living in the household. This cross-sectional, web-based survey was administered by the National Opinion Research Center (NORC) at the University of Chicago in English and Spanish.

Recruitment of parents across all Chicago neighborhoods for this wave of the VOCHIC Parent Panel Survey occurred through three mechanisms implemented by NORC: (1) the VOCHIC probability-based Parent Panel, (2) NORC’s probability-based AmeriSpeak panel, and (3) established online nonprobability survey panels. Responses from Chicago parents surveyed through nonprobability samples (i.e., convenience samples) were included to ensure sufficient sample size, which has been shown to be a cost-effective method to supplement probability-based samples [21,22]. This multi-component approach to recruitment has yielded representative and robust samples for other studies focusing on a broad variety of child and family health topics [23,24,25,26]. Respondents received a single compensation of USD 15 or USD 5 if they were first-time or repeat participants, respectively, as first-time respondents provided additional demographic information. Only one parent per household responded. A local IRB review assigned this survey exemption status and participants provided informed consent to participate by proceeding with the survey.

### 2.2. Cohort Demographic Measures and Outcome Measures

Parents provided demographic information by self-reporting. Household income was converted to three categories of the US federal poverty level (FPL) [27]: (1) low income, <100% FPL; (2) middle income, 100–399% FPL; and (3) high income, 400%+ FPL. Parent education was reported as (1) high school education or below, (2) some college or technical school education, or (3) a college degree or higher. Parent gender response options were female, male, and nonbinary/other gender. With differences in EPA and DHA intake by race and ethnicity previously documented in the United States [15,28], parents provided self-reported categories of their own race and ethnicity; these categories were included for this analysis in context of social constructs, not as indicators of causal roles in nutrient intake. Responses were combined into four groups: (1) Black, non-Latinx/Hispanic; (2) Latinx/Hispanic; (3) Other/Multi-race, non-Latinx/Hispanic; (4) White, non-Latinx/Hispanic.

Given the strong evidence for reduction in PTB from omega-3 PUFA intake, female respondents were also asked if they had any prior PTB (yes/no), defined as delivery prior to 37 weeks of gestation [29]. Also, the reported home or residence address was linked to each participant’s neighborhood Childhood Opportunity Index (COI) [30]. The COI uses census tract data to assign one of five levels (very low, low, moderate, high, very high) reflecting multidimensional opportunities for children’s healthy growth and development within that neighborhood. The COI is a composite score calculated from 44 individual factors (e.g., walkability, concentration of fast food restaurants, standardized test scores) within three primary domains (neighborhood environment, factors that impact health, educational opportunities) and has even been associated with outcomes in adults [31]. As an example, for families living in lower-COI neighborhoods, children had increased risk of obesity, emergency department visits, and mortality [32,33,34].

The primary outcome measures for the present study were parental average daily dietary intake of EPA and DHA. Subsequent analyses assessed the outcome of the combined daily intake of EPA+DHA.

### 2.3. Survey Weighting and Sample Probability

Survey responses were weighted to be representative of the population of Chicago parents based on Chicago parent population totals from the American Community Survey regarding age, sex, education, race and ethnicity, and Chicago Community Side (i.e., Central, North, Far North, Northwest, West, South, Southwest, Far Southwest). The combined probability and nonprobability sample weights were derived using small-area estimation methods [35,36]. These methods are frequently used by the US Census Bureau and national survey research organizations because of their efficiency and effectiveness [21,22].

### 2.4. Statistical Analysis

Demographic characteristics were summarized using survey-weighted proportions. Comparisons of intake between mothers versus fathers using *t*-tests were calculated and reported as the mean difference in intake of EPA and DHA. Multivariable linear regression and survey-weighted models evaluated parental characteristics associated with the combined EPA+DHA intake (mg/day). Individual models were developed for mothers and fathers because PTB was assessed only in female respondents. Otherwise, both models included the exposure variables of parent age, race, ethnicity, household income as percent of FPL, and reporting of DHA-containing supplement use (yes/no). Parent age was dichotomized as <35 years or ≥35 years based on the distribution of parent age categories to create sub-groups that were similarly sized. Due to collinearity with household income, parent education level was not included in analyses. For all parents together, pairwise comparisons estimated the mean difference in combined EPA + DHA intake across COI levels with high and very high levels grouped together. We used t-tests to test for mean differences. Analyses were performed in SAS software version 9.4 (SAS Institute, Cary, NC, USA) with α set at *p* < 0.05.

## 3. Results

**Figure 1:**
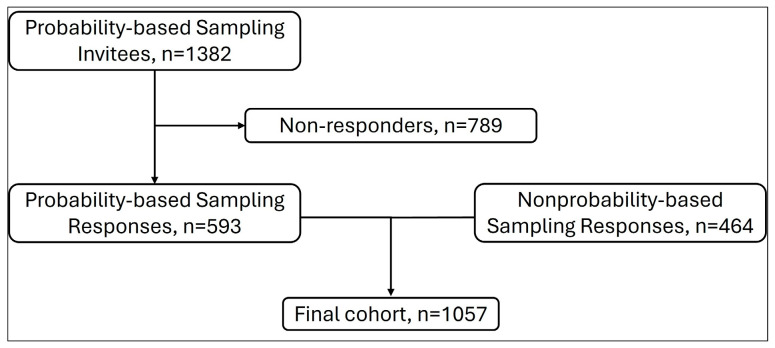
Flowchart showing responses from probability-based and nonprobability-based sampling.

Responses were collected from 1057 parents, including 593 parents from the probability-based sample and 464 from the nonprobability-based sample (Figure 1). The probability-based sample was from 1382 eligible invitees, yielding a survey completion rate of 42.9%; a survey completion rate for the nonprobability-based sample could not be calculated as these panels utilize opt-in online surveys (i.e., a denominator is not available).

Most participants (65.6%) were over 35 years of age at the time of the survey and were female (Table 1). Among female parents, 82.9% were ≤45 years of age and almost one-quarter reported having at least one prior PTB. Approximately one-quarter of parents reported using any DHA-containing supplement. The COI levels for most households were very low or low.

### 3.1. Parent Intake of EPA+DHA

Mothers reported significantly lower intake of EPA and DHA individually and combined EPA+DHA compared to fathers (Table 2). In multivariable regression models for mothers (Table 3), prior PTB and no use of a DHA-containing supplement were negatively associated with EPA+DHA intake. Black, non-Latinx/Hispanic race and ethnicity, as compared to White, non-Latinx/Hispanic, were associated with higher EPA+DHA intake. The household income categories 100 to 399% of FPL and 400% of FPL showed a positive association with EPA+DHA intake in a stepwise pattern as income increased, compared to households with an income <100% FPL.

The patterns of associations between race and ethnicity and EPA+DHA intake were different for fathers compared to mothers (Table 4). Paternal Latinx/Hispanic ethnicity and Other/Multi-race, non-Latinx/Hispanic ethnicity were associated with lower EPA+DHA intake. In contrast to mothers, household income was not associated with father’s EPA+DHA intake. However, as with mothers, no use of a DHA-containing supplement was associated with lower EPA+DHA intake for fathers.

### 3.2. EPA+DHA Intake by Childhood Opportunity Index

The mean parental EPA+DHA intake was highest for families in households with high/very high COI levels (Table 5a). For pairwise comparisons of estimated mean differences in parental EPA+DHA intake, households with high/very high COI levels reported significantly higher intake as compared to households with a low COI level (Table 5b). Households with high/very high COI levels also reported significantly higher parental EPA+DHA intake compared to households with a very low COI level (Table 5b).

Post hoc analyses to determine achieved power using the G*Power software version 3.1.9.6 revealed that, for all analyses presented in this manuscript, the achieved power to detect a medium effect size with alpha set at *p* < 0.05 ranged from 98% to 100%.

## 4. Discussion

This unique analysis reports a cross-sectional measure of parental EPA and DHA intake specifically in households with children in conjunction with other measures of children’s health potential. Specifically, these measures are preterm birth and the neighborhood factors reflected in the COI. Parental intake of EPA and DHA in households across a diverse, urban geography was considerably lower than the 250 mg/day that would be expected from consuming the amount of seafood recommended for American adults by the Dietary Guidelines for Americans [1]. Furthermore, when considering the magnitudes of significant differences in intake, with approximately 50–75 mg/d based on DHA supplement use and approximately 50 mg/d for different COI levels as examples, these indicate meaningful differences in eating habits and should not be considered small variations.

Current lower EPA+DHA intake by mothers in our survey was independently associated with a child born preterm in a past pregnancy even when accounting for other demographic characteristics. Early spontaneous preterm birth can stem from inflammatory processes [37,38]. Labor involves prostaglandin E2 from arachidonic acid (an omega-6 fatty acid which is generally proinflammatory). Omega-3 fatty acids and downstream lipid mediators may reduce preterm birth by countering the effects of inflammation from arachidonic acid that lead to preterm labor [3]. Preterm birth imposes substantial burdens on families, for both parents and children, including respiratory and neurodevelopmental morbidities as well as increased healthcare utilization for children [39]. For further context for parental intake reported in this study, a cohort of Norwegian women with a PTB rate ~5% had a median daily omega-3 PUFA intake greater than 400 mg from food sources alone [40]. DHA supplementation for pregnant women in clinical trials has lowered risk of PTB, and women with a lower DHA status based on red blood cell concentrations benefited the most from increased DHA intake through supplementation [3]. Yet women of reproductive ages in the United States have consistently consumed lower than recommended amounts of EPA and DHA [28]. In this study, the majority of mothers were of reproductive age. Thus, future pregnancies for women who reported prior PTB will be at risk of preterm delivery again, carrying risk of adverse health outcomes for those children born preterm. This is modifiable by increasing omega-3 fatty acid intake. Improving parental intake and reducing risk of PTB can directly improve the health of parents and their children. While pediatricians are charged with counseling parents about children’s eating habits, an intriguing consideration with these results is whether there is a role for pediatricians in directing parents to resources for their own nutritional counseling.

Across domains of household income and the COI, socioeconomic disadvantage (e.g., lower household income, lower COI level) was associated with lower parental intake of EPA+DHA. Neighborhoods categorized in lower COI have been associated with indicators of barriers to healthy eating, such as food deserts [17]. Our findings add new information regarding the COI as a potential indicator of specific aspects of a family’s diet quality, including at the level of nutrient intake. To consider addressing the lower intake for parents with a lower COI, besides seafoods, there are few good food sources of EPA and DHA. Importantly, adults eating vegetarian and vegan diets as well as those with an increased body mass index have reported lower EPA and DHA intake [41,42]. The Dietary Guidelines for Americans and the American College of Obstetricians and Gynecologists recommend 8–12 ounces of seafood per week for adults, including women who are pregnant or are going to be pregnant [1,4].

Seafood consumption can be accomplished safely through attention to potential environmental pollutants, even during pregnancy, with attention to the types of seafoods consumed [1,43]. Microplastics have been detected in marine food sources, yet current information suggests relatively low exposures to humans as the plastics remain isolated in animal tissues that are not consumed [44]. Another concern is of pesticides and heavy metals that enter waterways due to proximity to industrial processes [45]. These pollutants can concentrate in seafoods, yet this is not a globally uniform finding and the frequency of consumption can impact exposure [45]. In this context, daily seafood consumption is not a necessity to achieve recommended EPA+DHA intake. Still, ongoing attention and modeling of exposures through seafoods is warranted for safety monitoring [45]. While participants in this study lived in the midwestern United States, region-specific public health resources are available to help families make safe choices regarding types of seafoods ranging from locally caught lake fish to seafoods purchased at markets or grocery stores [4,46]. This is important because insufficient seafood consumption during pregnancy may have negative implications on children’s neurodevelopment [6]. Also, higher seafood intake by parents can increase their EPA and DHA intake and be a means for children to increase theirs as well [16,47,48]. While fresh seafood is generally more expensive than many protein sources, canned and frozen seafood may be more affordable and PUFAs are similarly bioavailable in them compared to in fresh seafoods. There are products to which DHA has been added, e.g., milk and yogurt, that are available in some markets including the midwestern region of the United States. Aside from these types of food fortifications, for which the amount provided would be noted on the food label, supplements that contain DHA or EPA and DHA remain the main other source to improve intake. Neighborhood-level interventions to educate communities about the benefits of, choices of, and purchasing options for seafoods could reduce barriers and improve intake.

We detected different associations with race and ethnicity depending on whether mothers or fathers responded, which has been observed in other reports of dietary intake [49]. This affirms the concept that dietary intake of omega-3 fatty acids by any individual may be shaped by multiple social roles, including but not limited to sex and race or ethnicity. While some reports of dietary patterns assess intake based on sex and race or ethnicity separately [50], this justifies using statistical models that consider intersectionality and test for differences using multiple social categories in the same analysis [51]. The differential associations with race and ethnicity categories amongst parents may more directly reflect cultural traditions and differences which are important influences on eating patterns, as emphasized by the Dietary Guidelines for Americans [1].

Parents report intentionally improving their own diet quality so that their children eat heathier as well [52]. We focused on parental EPA and DHA intake based on the evidence that parents are an important influence on their child’s eating habits and are also in control of food that comes into the home [53]. Simultaneous assessment of intake in both parent and child is an important future direction for research because other factors may influence children’s eating behavior. Examples include parents showing enthusiasm for healthy eating or deciding not to watch TV during mealtime [12,54], influences outside of the immediate family such as peers and caregivers [55,56], and food advertisements [57,58,59]. We did not have the opportunity in this study to examine child intake.

With attention to parental intake as relevant to the health of children, it is also important to note that achieving sufficient PUFA intake can directly and favorably impact the health of parents/adults. Increasing intake of EPA and DHA can reduce cardiovascular events and inflammatory conditions including inflammatory bowel disease and also preserve cognitive function in adults [7,8,60]. Protective mechanisms likely operate through pathways involving PUFA-derived mediators, which can be influenced by dietary intake [61].

While the FFQ utilized in this survey was validated in pregnant women, it assesses intake of foods in the American diet that contribute most to EPA and DHA consumption. FFQs assessing PUFA intake have consistently been validated and show good correlation with EPA and DHA blood concentrations in both men and women, whether pregnant or not, and across distinct geographic locations and various medical diagnoses [19,62,63,64,65]. Therefore, we assume that these same foods are appropriate to assess in non-pregnant adults in the United States.

Eggs and chicken are also good sources of DHA. A possible limitation of our study was that the survey was distributed in Spring and Summer 2022, and an outbreak of avian flu in the United States beginning in February 2022 was associated with increased prices of eggs and poultry [66]. Also in the United States, the COVID-19 pandemic was associated with decreased consumption of seafoods, eggs, and poultry [67]. These events could have decreased EPA and DHA intake. Another limitation is that we asked that only one parent per household complete the survey. Eating patterns between parents in the same household may be related [16]; however, averaging two reports from each parent might have better represented households with two parents. The COI levels for households in this survey were predominantly low or very low in contrast to national distributions for households showing even distribution across the five levels of the COI [68]. Therefore, our results cannot be generalized to all families in the United States.

Although Chicago is adjacent to the Great Lakes, global comparisons show that American adults have some of the lowest EPA and DHA consumption compared to other countries based on blood biomarkers [69]. This reflects different eating habits including but not limited to seafoods [70]. Other dietary habits such as supplement use may also be relevant to optimizing household intake. As an example, the prevalence of using cod liver oil which has high concentrations of omega-3 fatty acids ranges widely between countries [71,72]. From a global perspective, developing affordable plant-based sources of omega-3 fatty acids may increase opportunities to improve intake, but these options are not yet widely available [73].

The strengths of this study stem from the novel consideration that parental dietary intake might reflect health potential of their children. Also, the FFQ used in this study has been previously validated with blood biomarkers of omega-3 fatty acids [19]. Use of survey weighted data facilitated the data being representative of parents in this large, urban city with diversity in socioeconomic status. However, a limitation in generalizability of the findings is that households were in neighborhoods primarily categorized as having a very low or low COI. Additional limitations include the cross-sectional nature of the survey which might be relevant to seasonal availability of foods, inability to quantify intake specifically from supplements, and that we were not able to assess specific eating locations for meals, which can impact diet quality [74].

## 5. Conclusions

Intake of EPA and DHA by parents across a diverse, urban population did not meet recommendations for most of the respondents. Improving intake stands to benefit the public’s health in quantifiable ways, with a reduction in preterm birth rates as an example. Importantly, our results support the concept that efforts aimed at improving parental EPA and DHA intake may be most effective by accounting for personalized, multidimensional influences on household food choices. Examples of such efforts include dietary guidelines at national and local levels, as well as healthcare institutions and their staff who provide dietary counseling for families. Future work should include assessment of both parents in two-parent households, also addressing families with different structures, in conjunction with children to define and address barriers to sufficient EPA and DHA intake for all family members.

## Figures and Tables

**Table 1:** Sociodemographic characteristics for 1057 Chicago parents reporting eicosapentaenoic acid and docosahexaenoic acid intake.

Respondent Characteristics	n = 1057
Age category	
18–25 years	106 (3.8)
26–35 years	343 (30.6)
36–45 years	426 (41.0)
46–55 years	151 (20.7)
56–65 years	26 (3.5)
Over 65 years	5 (0.4)
Gender	
Female	806 (59.5)
Male	245 (40.5)
Nonbinary	2 (<1)
Race and ethnicity	
Black, non-Latinx/Hispanic	250 (21.6)
Latinx/Hispanic	337 (36.3)
Other/Multi-race, non-Latinx/Hispanic	106 (10.0)
White, non-Latinx/Hispanic	364 (32.1)
Highest level of education	
High school or below	194 (34.5)
Some college or technical school	292 (24.4)
College graduate or above	563 (41.1)
Annual household income, US dollars	
Less than 100% of FPL	155 (17.3)
100 to 399% of FPL	493 (47.4)
400% or greater than FPL	409 (35.3)
Number of children <18 years in household	
1	498 (49.9)
2	385 (33.4)
3	124 (10.8)
≥4	50 (5.9)
Prior preterm birth *	146 (24.1)
Any DHA supplement use	302 (25.3)
Childhood Opportunity Index	
Very low	508 (46.1)
Low	295 (27.2)
Moderate	136 (15.2)
High/very high	118 (11.5)

Values shown are n (survey-weighted proportion); FPL, federal poverty level; DHA, docosahexaenoic acid. Missing: gender, n = 4; education, n = 8; supplement use, n = 4. * female respondents.

**Table 2:** Reported EPA and DHA intake by Chicago parents.

	Mothers n = 806	Fathers n = 245	Mean Difference in Intake for Mothers	*p*-Value *
EPA intake, mg/d	47.7 (2.1)	57.6 (3.8)	−10.0 (4.4)	0.02
DHA intake, mg/d	87.5 (3.5)	105.2 (6.2)	−17.7 (7.1)	0.01
Combined EPA+DHA, mg/d	135.7 (5.6)	162.8 (10.0)	−27.1 (11.4)	0.02

EPA, eicosapentaenoic acid; DHA, docosahexaenoic acid. Values are mean (SE). * T-tests were used to test for mean differences. Intake for 2 parents self-identifying as nonbinary: EPA: 221 mg, 27.5 mg; DHA: 329 mg, 59.5 mg; combined EPA+DHA: 550 mg, 87 mg.

**Table 3:** Associations between sociodemographic factors and combined EPA+DHA intake (mg/d) for Chicago mothers (n = 654).

Variable	Coefficient (EPA+DHA Intake, mg/d)	95% CI	*p*-Value
Prior preterm birth	−24.4	−48.5, −0.2	0.048
Maternal age ≥ 35 years *	−3.1	−29.6, 23.4	0.82
Black, non-Latinx/Hispanic **	41.7	6.1, 77.4	0.02
Latinx/Hispanic **	−4.7	−34.1, 24.7	0.75
Other/Multi-race, non-Latinx/Hispanic **	28.2	−24.0, 80.5	0.29
Household income 100 to 399% FPL ^†^	29.0	1.2, 56.8	0.04
Household income 400% or greater FPL ^†^	57.1	20.5, 93.8	0.002
No use of DHA-containing supplement	−48.3	−77.3, −19.3	0.001

EPA, eicosapentaenoic acid; DHA, docosahexaenoic acid; FPL, federal poverty level. A multivariable linear regression and survey weighted model evaluated associations between the exposure variables and the outcome of EPA+DHA intake. All variables were included together in this single model. Of the 806 female respondents, those with missing responses for preterm birth or any covariate were not included in the model (n = 152). * Age was dichotomized in the model due to small weighted proportions at either end of the range. ** Reference group is White, non-Latinx/Hispanic. ^†^ Reference group is household income <100% of federal poverty level.

**Table 4:** Associations between sociodemographic factors and EPA+DHA intake (mg/d) for Chicago fathers (n = 245).

Variable	Coefficient (EPA+DHA Intake, mg/d)	95% CI	*p*-Value
Paternal age ≥ 35 years *	15.0	−27.1, 57.1	0.48
Black, non-Latinx/Hispanic **	−10.6	−70.2, 49.1	0.73
Hispanic **	−52.1	−101.0, −3.3	0.04
Other/Multi-race, non-Latinx/Hispanic **	−58.5	−111.6, −5.4	0.03
Household income 100 to 399% FPL ^†^	9.0	−63.2, 81.1	0.81
Household income 400% or greater FPL ^†^	2.2	−73.3, 77.6	0.96
No use of DHA-containing supplement	−73.0	−117.0, −29.0	0.001

EPA, eicosapentaenoic acid; DHA, docosahexaenoic acid; FPL, federal poverty level. A multivariable linear regression and survey weighted model evaluated associations between the exposure variables and the outcome of EPA+DHA intake. All variables were included together in this single model. * Age was dichotomized in the model due to small weighted proportions at either end of the range. ** Reference group is White, non-Latinx/Hispanic. ^†^ Reference group is household income <100% of federal poverty level.

**Table 5:** Parental EPA+DHA intake (mg/d) across Childhood Opportunity Index (COI) categories. (**a**) Mean EPA+DHA intake (mg/d) by COI category; (**b**) pairwise comparisons for the estimated mean differences in EPA+DHA intake (mg/d) across COI categories.

**(a)**				
**COI category**	Very low n = 508	Low n = 295	Moderate n = 136	High/Very high n = 118
EPA+DHA intake *	139.6 (7.2)	131.7 (7.6)	167.0 (17.8)	188.9 (19.3)
**(b)**				
**COI group comparisons**			Mean difference (SE)	*p*-value **
**Low − Very low**			−7.9 (10.5)	0.45
**Moderate − Very low**			27.4 (19.2)	0.15
**Moderate − Low**			35.3 (19.3)	0.07
**High/Very high − Very low**			49.3 (20.6)	0.02
**High/Very high − Low**			57.2 (20.7)	0.006
**High/Very high − Moderate**			21.9 (26.2)	0.4

EPA, eicosapentaenoic acid; DHA, docosahexaenoic acid. * Values are mean (SE). ** T-tests were used to test for mean differences.

## Data Availability

The data presented in this study are available on request from the corresponding author. Data are not publicly available.

## Supplementary Materials

The following supporting information can be downloaded at https://www.mdpi.com/article/10.3390/nu17203277/s1. Supplemental Material. Summary of survey questions adapted from the original food frequency questionnaire and used to assess parental intake of eicosapentaenoic and docosahexaenoic acids.

## Abbreviations

The following abbreviations are used in this manuscript:

COI	Childhood Opportunity Index
DHA	Docosahexaenoic acid
EPA	Eicosapentaenoic acid
FFQ	Food frequency questionnaire
FPL	Federal poverty level
NORC	National Opinion Research Center
PTB	Preterm birth
PUFA	Polyunsaturated fatty acid
VOCHIC	Voices of Child Health in Chicago
