# Supplementary material for: Parental Intake of Eicosapentaenoic and Docosahexaenoic Acids in a Diverse, Urban City in the United States Is Associated with Indicators of Children’s Health Potential
Source: Nutrients. 2025 Oct 18;17(20):3277. doi: 10.3390/nu17203277 (PMC12566695; doi:10.3390/nu17203277)
Supplement: Supplementary file 1 [file nutrients-17-03277-s001.zip › nutrients-3849661-supplementary.pdf]

Supplemental material. Summary of survey questions adapted from the original food frequency questionnaire[1] and used to assess parental intake of eicosapentaenoic and docosahexaenoic acids.

Participants report their usual intake and supplement use for the following items:

1. **High Omega-3 Fish.** This category includes: salmon, sardines, mackerel, bluefin tuna, herring, whitefish, smoked cisco, pollock, bluefish.
   → Record number of 3-oz servings consumed per month.
2. **Medium Omega-3 Fish.** This category includes: trout, canned tuna (6-oz can), flounder, snapper, bass, perch, redfish, swordfish, catfish, grouper, rockfish, mussels, sole, shark, drumfish.
   → Record number of 3-oz servings consumed per month.
3. **Low Omega-3 Seafood.** This category includes: cod, haddock, fish patties/squares, fish sticks, scallops, shrimp (14 medium), oysters, mullet, sturgeon, clams, crab, crayfish, lobster, pompano, carp, pike.
   → Record number of 3-oz servings consumed per month.
4. **Liver Consumption**. This category includes: chicken, turkey, or beef liver.
   → Record number of 3-oz servings consumed per month.
5. **Egg Yolks.** This category includes all yolks, including those used in cooking and baking.
   → Record total number consumed per week.
6. **Poultry Intake.** This category includes: chicken, turkey, or other poultry (excluding liver).
   → Record number of 3-oz servings consumed per week.
7. **Any omega-3 supplement use.**
   - → Record yes or no.

Calculation Method to derive total daily eicosapentaenoic acid (EPA) and docosahexaenoic acid (DHA) intake (mg/d) from food sources:

1. Assign average EPA and DHA values to each food category using the USDA Nutrient Database (Release 14, 2002).
   - High Omega-3 Fish: ~22 mg DHA, ~14 mg EPA per 3-oz serving
   - Medium Omega-3 Fish: ~10 mg DHA, ~5 mg EPA per 3-oz serving
   - Low Omega-3 Seafood: ~5 mg DHA, ~6 mg EPA per 3-oz serving
   - Liver: ~7 mg DHA, ~2 mg EPA per 3-oz serving
   - Egg yolk: ~3 mg DHA, ~0.25 mg EPA per yolk
   - Poultry: ~5 mg DHA, ~3 mg EPA per 3-oz serving
2. Multiply reported intake by assigned EPA and DHA values to obtain totals for each food category.
3. Convert weekly or monthly totals to daily averages (divide weekly intake by 7; monthly by 30.4).
4. Add intake values from all food categories to determine total daily EPA and DHA intake.

Reference.

1. Christifano DN, Crawford SA, Lee G, et al. Utility of a 7- question online screener for DHA intake. *Prostaglandins Leukot Essent Fatty Acids*. 2022;177:102399.
